# Roughness integration across fingers within compared with across the hands

**DOI:** 10.1038/s41598-024-83308-4

**Published:** 2024-12-30

**Authors:** Roberta D. Roberts

**Affiliations:** School of Psychology, University of Birmingham, Birmingham, B15 2TT UK

**Keywords:** Active touch, Roughness, Texture, Multidigit, Bilateral, Psychology, Human behaviour

## Abstract

Feeling a texture typically involves sliding the fingers of a hand across that surface or rubbing the surface between the thumb and another digit. Texture signals appear to be integrated across the digits of a hand with perceived roughness at one finger swayed in the direction of texture touched by another finger of the same hand. To date, one study has reported similar integrative effects when the pairs of digits belong to different hands. This contrasts with observations from studies of tactile detection and tactile attention where the patterns of interactions between the digits depend on whether the digits belong to the same hand or not. The present experiments revisit the question of hand identity on multidigit roughness perception using two interval forced choice (2IFC) discrimination and single interval absolute magnitude estimation (AME). Pairs of tactile gratings were actively touched using the thumb and index fingers from the same or different hands. Attention was directed towards roughness at the thumb and index finger sensations were to be ignored. For both discrimination and ratings tasks, roughness perceived at the thumb varied with the textures touched by the index finger suggesting integration of roughness cues from the two digits. This integration occurred despite differences in the two tasks such as working memory requirements. Notably, roughness signals were integrated when originating in pairs of digits on the same hand but not when from different hands. These findings add to a body of evidence based on experiments using different stimuli and tasks, suggesting that hand identity affects interactions across the digits.

## Introduction

In touch, feeling the texture of an object seldom involves the use of only a single finger. Rather, in unconstrained settings, the textures of objects are actively explored using sweeps or rubbing motion of multiple digits, in some cases including fingers and opposed thumb^1^. Despite this, there is limited knowledge at present about whether and how the brain might combine this information from multiple touch sources to generate an overall sense of the texture of touched surfaces. This is particularly true for somatosensory signals from multiple fingers across the two hands and is the issue that is explored in the present work.

There is some evidence that the perception of tactile roughness on one finger can be altered by roughness signals originating from other digits within the same hand^2–4^. Verrillo and colleagues^2^ used an absolute magnitude estimation (AME) task to measure the perceived roughness of sandpapers presented to the index finger, thumb or to both digits. On each trial of their two-digit conditions the same grade of sandpaper was felt by both digits and participants’ attention was undirected. While Verrillo and colleagues found no difference in magnitude estimates of sandpaper roughness when using either the index finger or thumb alone, they did find significantly higher values (indicating greater perceived roughness) were assigned when both digits were used simultaneously. The authors suggested that these findings could be accounted for by a form of spatial summation over the digits.

This integration account is complemented by the findings of experiments measuring roughness discrimination of sandpapers^3^ and of dot patterns embossed on paper^4^. In these studies, roughness perception was measured using a discrimination task with attention directed towards a cued, target digit and away from a distractor digit. Under such conditions the coarseness of surfaces touched by the unattended (distractor) digit systematically influenced the perceived roughness of the surface touched by the cued (target) digit. Discrimination of the roughness at the target digit was biased towards smoother judgements when a finer texture was touched by the distractor digit and towards rougher judgments when a coarser distractor texture was touched. This suggests that, when touching textures using a pair of digits from the same hand, it is difficult to restrict attention to the roughness experienced by just one of those digits.

Interestingly, the pattern of interaction between the digits on a single hand has been found to be affected by the separation of the digits^3^. Digits occupying neighbouring locations on the hand showed interaction patterns consistent with the integration of roughness signals. However, when touching with more distant digits on the same hand there was no such pattern of interaction but simply reduced overall roughness sensitivity at the attended digit. One interpretation of the interaction between the fingers in roughness discrimination is that it reflects cortical integration. Signals from neighbouring digits, whose cortical receptive fields overlap more than non-adjacent digits, might be expected to show more integration. On this view, it might be expected that there should be no integration of roughness signals originating from widely separated sites on the body’s surface, for example between digits on different hands. However, interactions between roughness signals from the thumb and index finger have been observed when using both digits to slide simultaneously across differing pairs of sandpapers^5^. This integration was evident both when the thumb and index finger were adjacent digits on the same hand and when they were on different hands. This finding suggest integration of roughness signals may not be caused simply by cortical proximity reflecting proximity on the body’s surface but may also involve cortical areas integrating information from different sides of the body.

A notable feature of the work showing roughness perception across the hands^5^, but also some of those studies showing within hand interactions^2,3^, is the use of sandpaper surfaces as stimuli. The multidimensional nature of such surfaces, containing a range of spatial frequencies and unknown spatial distribution of the sharp constituent elements, make it difficult explore the psychometric properties relating stimulus features to perceived roughness. In addition, the repeated contact with these abrasive surfaces required for psychophysical measurement is likely to degrade both the test surfaces and the skin, potentially altering contact dynamics and perception of the surfaces while touching.

Using square wave gratings, the present pair of experiments revisit the question of whether hand identity affects interdigit interactions in roughness perception. In contrast to abrasive surfaces, the spacing of square wave elements are better controlled, allowing clear contrasts between fine (few hundred microns) and coarse (on the order of thousands of microns) surfaces. In the present work, the use of such stimuli made possible exploration of the psychometric functions underlying one-handed and two handed texture discrimination, as well as extension of roughness magnitude estimation to include fine-grained target-distractor differences in stimuli. Experimental designs previously shown to elicit interactions between pairs of digits, two interval forced choice (2IFC) discrimination and single interval absolute magnitude estimation (AME), were matched across various aspects of experimental design including attentional focus and target-distractor differences in roughness. Complementary but different approaches to measuring roughness perception were employed to both replicate and extend previous work as well as increase generalisability of findings across paradigms. To this end, 2IFC and AME tasks were used to ask whether roughness perception at an attended digit is affected by roughness at a distractor digit and whether the magnitude of any interaction is the same in one handed and two handed touch. In both tasks attention was directed towards a target digit (the thumb) and away from the distractor digit (the index finger). Given that interdigit interactions are, in part, thought to arise in cortical regions processing signals from one side of the body, but also show some evidence of spanning the hands, interference was anticipated both when the target and distractor digits were on different hands compared with the same hand. Specifically, both roughness discrimination performance and magnitude ratings for target surfaces were expected to vary systematically with distractor roughness (reflecting signal integration effects) in both one-handed and two-handed touch conditions.

## Experiment 1: roughness discrimination

### Method

#### Participants

Seventeen participants took part in this experiment. All were classified as strongly right-handed (laterality index of 100) using a subset of the Edinburgh Handedness Inventory^6^. Normal sensation in their hands and normal, or corrected-to-normal, vision was reported by all participants. Their ages ranged from 19 to 38 years, with a mean age of 24 ± 4 years. Twelve of the participants were female. All gave informed consent and received £18 for their participation. This work was conducted in accordance with APA standards for the ethical treatment of subjects and with the approval of the University of Birmingham’s Life and Health Sciences Ethical Review Committee (ERN_08-186).

#### Stimuli

**Fig. 1:**
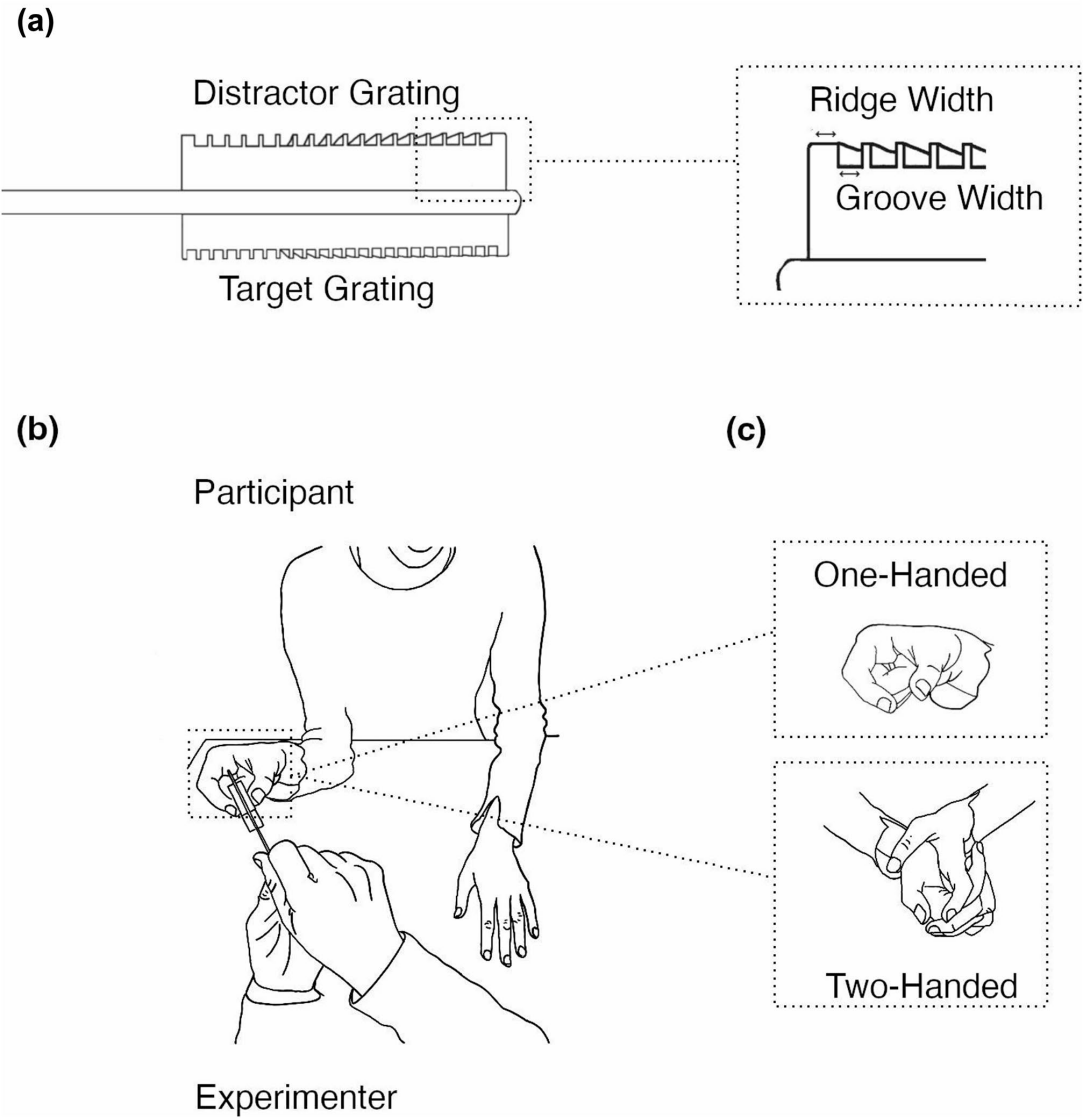
The grating stimuli and experimental setup used in experiments 1 and 2. Grating stimulus pairs consisting of two square wave gratings mounted either side of a single card as shown in (**a**). Participants attended to the target grating, touched by their right thumb while the experimenter held the card in place (**b**). Distractor gratings were touched simultaneously by the right index finger (in one-handed conditions) or left index finger (two-handed conditions). Hand postures are shown in (**c**).

Participants were asked to judge the roughness of a set of square wave gratings milled from blocks of Tufset Polyurethane (http://www.bayplastics.co.uk/tufset.htm).The groove widths (GW) used were as follows: 330, 880, 1120, 1160, 1200, 1240, 1360 and 1520 μm. The ridge width (RW) for each grating was 400 μm, except for the 330 μm GW grating, where the ridge width was 100 μm. See Roberts et al.^7^ for previous use of these square wave gratings in 2IFC roughness discrimination. A double-sided grating stimulus set, i.e. a grating pair, was created by attaching two gratings, back-to-back, to a 1.5 mm thick piece of card (see Fig. 1a). The combined width of each double-sided grating pair was 13.5 mm. A back-to-back arrangement of texture pairs, requiring opposed finger and thumb exploration, was used in three^2,3,5^ previous studies showing roughness interactions between the digits. The opposed thumb and index finger exploration (precision touch) may be considered as analogous to precision grip that is commonly used take hold of and manipulate objects and has recently been observed during texture exploration^1^.

#### Design

The experiment consisted of 180 trials. Each trial comprised two observation periods (intervals) with a grating pair (see Fig. 1a) presented in each interval. During the first interval, a grating pair was presented to the participant who used their thumb and index finger to stroke the grating pair (see Fig. 1b). After the stroking action was completed, a second grating pair was similarly presented to and stroked by the participant. The interval between contacts was between approximately 1 and 2 s. The digits used to touch the stimuli belonged to the same hand (one-handed) in half of the trials and different hands (two-handed) in the other half (see Fig. 1c). In each interval, participants were directed to focus on the roughness of the (target) grating touched by their thumb, while ignoring the roughness of the (distractor) grating touched by their index finger. At the end of each trial, participants reported which of the two target gratings, touched using their thumb, felt rougher. Previous studies of selective attention in vibration^8^ and texture^5^ perception have used the thumb as the attended digit with the index finger as a distractor. Given that index fingers, along with the middle digit, show evidence of being specialized for fine analysis during active touch search^9^, the use of index fingers distractor surfaces in the present study was intended to maximise the chance of finding distractor effects.

Within a trial, one of the target gratings was coarser (1200 μm) and the other finer (1160 μm) in groove width. The same pair of target gratings (1200 vs. 1160 μm) was used on every trial. Previous work with square wave gratings has shown a monotonic increase in perceived roughness with increases in spatial period and groove width, and to a lesser extent with decreases in ridge width^10–13^. Consequently, the 1200 μm target was expected to be perceived as the rougher of the two gratings in the absence of a distractor.

Distractor gratings differed in groove width from the target gratings with which they were paired. In one interval of each trial there was a 40 μm difference in groove width between the target and distractor of the grating pair. This grating pair functioned as the *standard stimulus*. In the other interval of the same trial, differences between the target and distractor of the grating pair varied between 40 and 880 μm. This grating pair functioned as the *comparison stimulus*. Half of all trials had comparison stimuli with the finer target (1160 μm) and were labeled as “comparison-finer”. The remaining trials featured comparison stimuli with the coarser target (1200 μm) and were designated “comparison-coarser.” The specific combinations of gratings constituting standard and comparison stimuli are depicted in Table 1. Varying the groove width of comparison distractors on the index made it possible to examine how effectively participants were able to focus on the roughness of targets on their thumb as well as the nature of any interaction between target and distractor digits. With complete suppression of distractor input, performance would not be expected to vary with distractor groove width. In contrast, interactions between target and distractors varying systematically with the comparison groove width would suggest integration of roughness between the digits.

Each combination of conditions; number of exploring hands (one-handed versus two-handed), varying-interval (comparison-finer versus comparison-coarser) and the different distractor groove widths, was presented 10 times. The presentation order of the standard and comparison stimuli and coarser and finer targets within a trial was randomised across trials. One-handed and two-handed conditions were presented in separate blocks.

**Table 1:**
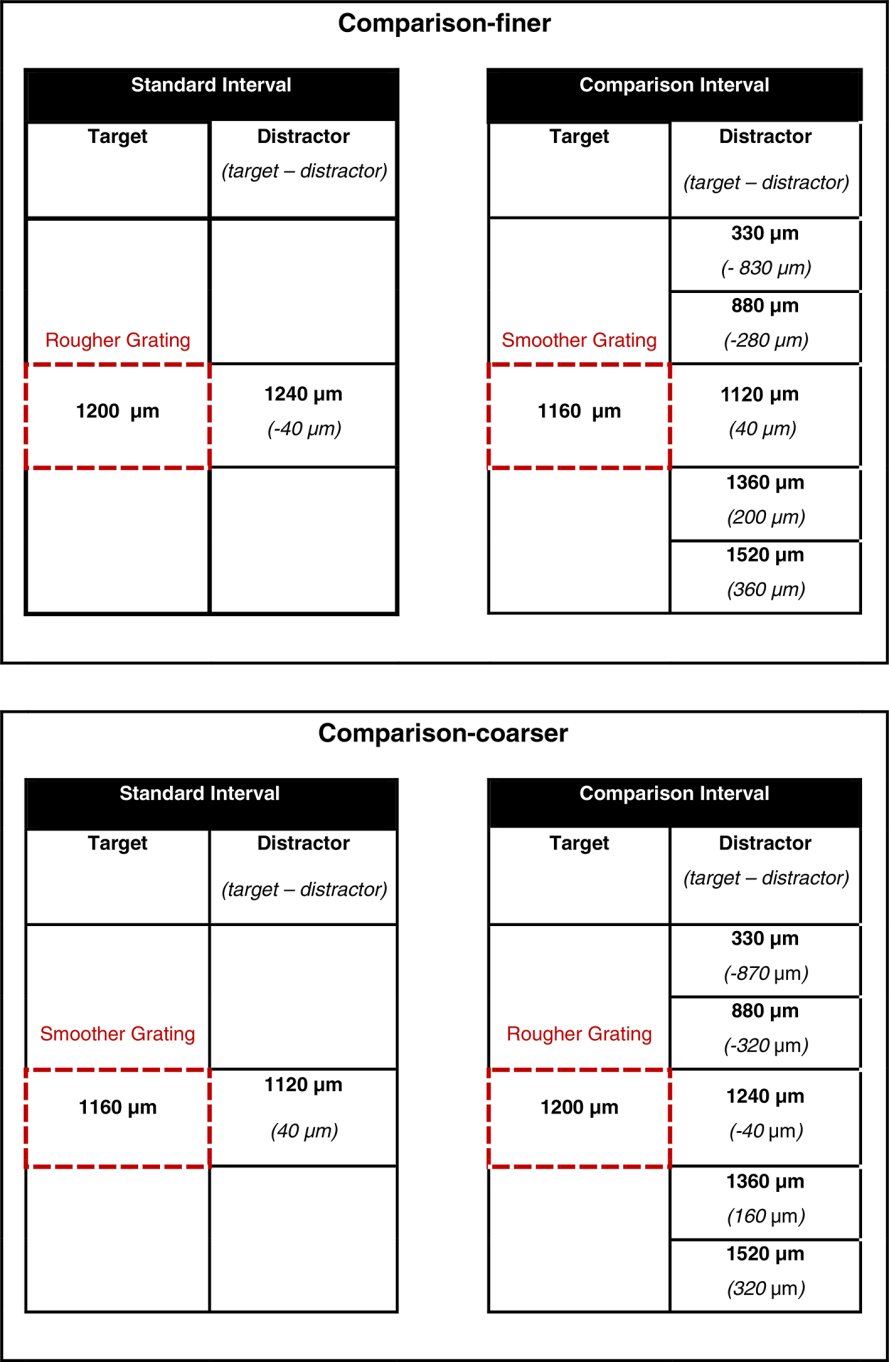
The combinations of stimulus groove width used to create standard and comparison intervals in the comparison-finer and comparison-coarser conditions. Each bold number is given in microns and represents the groove width of a grating surface. Values in brackets represent the groove width difference between target and distractor surfaces. Each experimental trial consisted of a standard and comparison interval with presentation of grating pairs set appropriate to the experimental condition. The order of the intervals in a trial was randomly determined in advance of testing.

#### Procedure

The participants sat across a table from the experimenter with their elbows supported by the table and their hands positioned towards the experimenter (Fig. 1b). They wore a blindfold and ear enclosing headphones (Sennheiser pxc 300) playing white noise at 52 dB to prevent them seeing the grating surfaces or hearing any auditory cues generated by contact between the digits and the stimuli. Participants were asked to use a sliding action away from the experimenter to slide their thumb and opposed index finger at a steady, comfortable, speed over the surfaces of grating pairs. They were directed to ensure that, while touching each grating pair, their digits maintained simultaneous contact with both gratings, moving along them in unison with equal force applied to each surface. Participants were encouraged to move at a self-selected, comfortable scanning speed with the consequence that contact times varied between participants. Only one unidirectional (towards the body) scan of the surfaces of the grating pair was permitted. Additionally, participants were asked to focus on the roughness sensations experienced at their thumb and ignore the roughness sensation from their index finger.

Each trial began with the experimenter positioning the first grating pair between the participant’s outstretched digits. Following a verbal cue from the experimenter, the participant brought their digits together contacting the gratings and then used the hand/s to slide the digits over the surfaces of the gratings. The grating pair was removed, and the procedure repeated approximately 1 to 2 s later with a second pair of gratings. The participant then reported which of the two target gratings touched by their thumb was rougher. The experimenter utilized visual and proprioceptive feedback, specifically monitoring for twisting of the stimulus card during exploration, to provide verbal feedback to participants regarding the application of equal and opposite, aligned normal force at each surface. A practice session of between 9 and 13 trials was run in which the experimenter gave training with feedback on both the movement and roughness discrimination elements of the task. In the data collection phase of the experiment, feedback was only given on the movement aspect of the task.

One central aim of the experiment was to examine the effect of hand identity on the ability to filter out sensory inputs from an irrelevant digit. To achieve this, roughness discrimination was measured in both one-handed and two-handed contact conditions. In the one-handed condition, the right thumb was used to explore the target grating, while the corresponding distractor grating was explored by the right index finger. In the two-handed condition, the target grating was also explored using the right thumb but the accompanying distractor was explored using the left index finger. In both conditions, a pinch grip posture was adopted, where the thumb opposed the index finger. In the one-handed condition the remaining digits were curled into the palm of the hand. In the two-handed condition the remaining fingers of each hand were interleaved to assist unified movement of the two digits contacting the gratings. These hand postures are shown in Fig. 1c.

Participants practiced performing both the precision grasp sliding movements and roughness discrimination at the start of the experiment. Practice trials were carried out using grating pairs where both the target and distractor gratings had groove widths of either 1280, 1200, or 1360 μm on both sides. Practice concluded after 9 trials, 3 pairings of each of the practice grating pairs with the others. For some participants practice sessions were extended until they were able to scan the stimuli in the manner requested. The maximum number of trials in a practice session was 13.

The main experiment was conducted over two sessions each lasting around 50 min and separated by between 1 and 3 days. One-handed and two-handed conditions were tested in separate sessions involving 90 trials each. Performance in the one-handed condition was measured first in 7 participants and the two-handed condition first in 10 participants. Rest breaks were taken approximately every 10 min or on request.

#### Data analysis

**Fig. 2:**
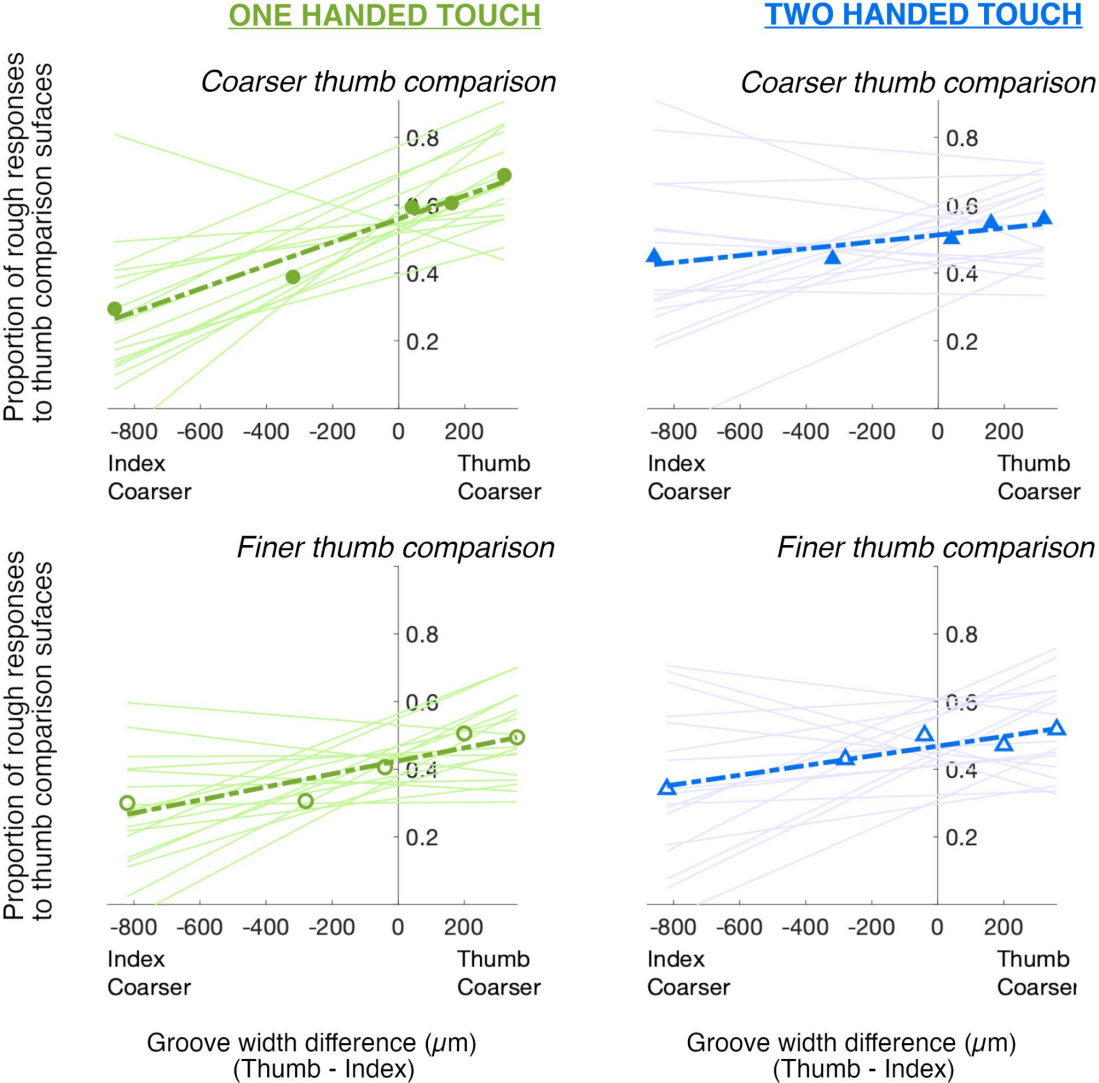
The proportion of trials where the target in the comparison interval was judged as the rougher of a pair of target gratings (1200 versus 1160 μm groove width). Green circles on the left side of the figure show mean data in one-handed touch. Two-handed touch performance is shown by blue triangles on the right side. Data in comparison-coarser conditions (1200 μm target paired with varying distractors) are shown as filled symbols while those in comparison-finer (1160 μm target paired with varying distractors) conditions as clear symbols. Thin continuous lines indicate linear functions fitted to the data of individual participants. Functions fitted to group mean data are shown as bold, dashed lines.

**Fig. 3:**
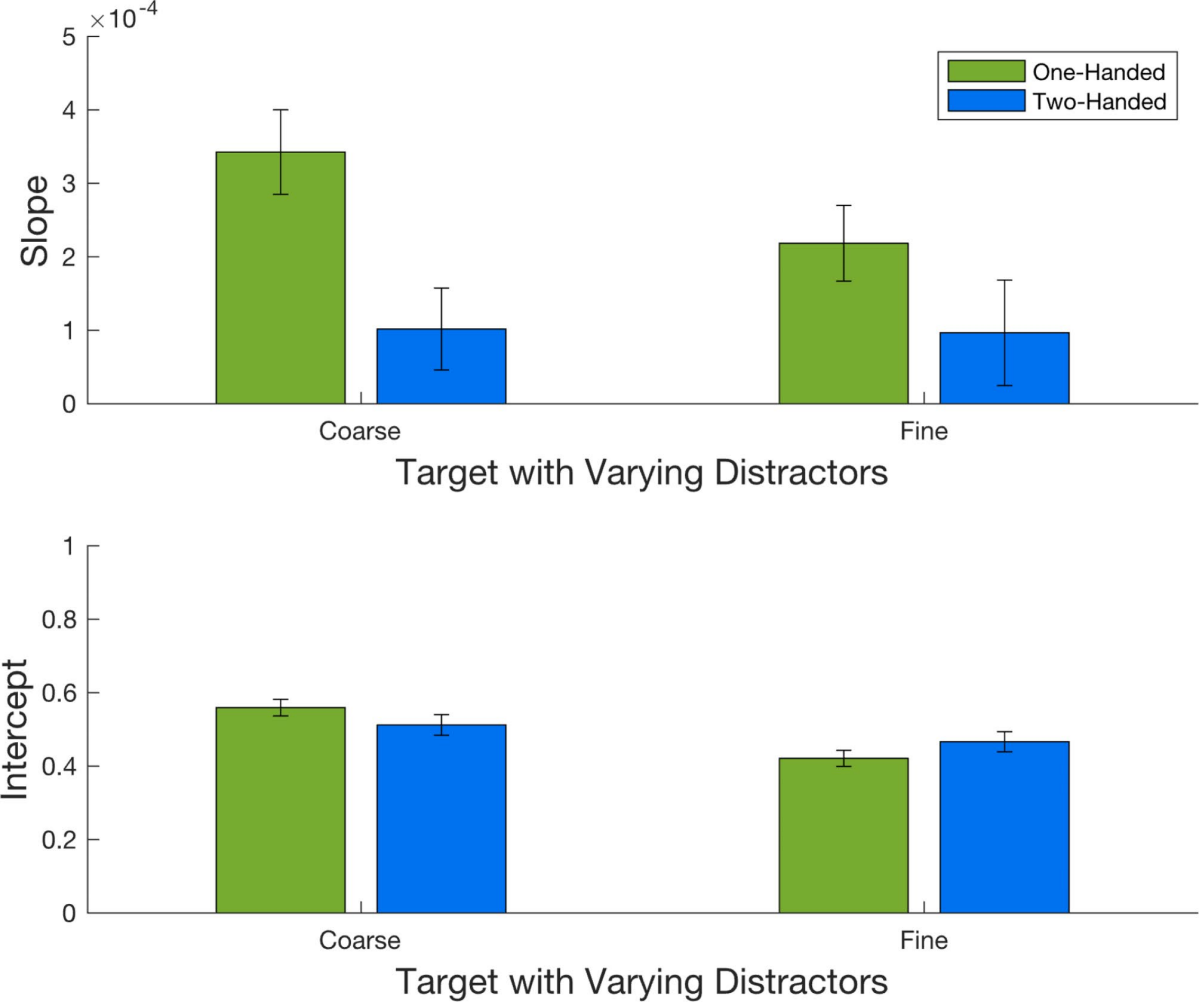
The mean slopes (top graph) and y-0 intercepts (bottom graph) of linear functions fitted to the data of individual participants. One SE is shown.

The proportion of responses where the target in the comparison interval was judged as rougher was calculated for each distractor groove width. A linear function was fitted across the distractor groove widths separately for each varying-interval (comparison-finer and comparison-coarser) and number of hands (one-handed and two-hands) of each participant with the slopes and intercepts optimized using a linear least-squares method (Matlab 2024). Single distractor outlier values (3 in the one handed and 2 in the two-handed conditions) were removed from the comparison-finer data to improve curve fits. Functions fits were better in one-handed (median r^2^ = 0.701) than two-handed (median r^2^ = 0.463) conditions. Linear functions fitted to the mean and individual data are shown in Fig. 2. Slope and intercept values are shown in Fig. 3.

Effects of distractors on roughness discrimination of target gratings were examined using a repeated measures Analysis of Variance (ANOVA) of the slopes and intercepts of linear functions fitted to the data of individual participants. The number of hands (one-handed or two-handed) used to touch the stimuli and the varying-interval (comparison-finer or comparison-coarser) were used as within-subjects factors.

## Results

The proportion of rougher discrimination responses to targets in comparison intervals can be seen as filled and open symbols in Fig. 2. Responses to targets varied with distractor groove width, indicating participants were not able to filter out roughness sensations from the distractor digit. Complete filtering out of distractors would have been evidenced by a zero slope in the linear function describing both individual and group data. Instead, Fig. 2 shows increased rougher judgments for targets paired with distractors of larger groove widths.

### Slope analysis

The slopes of functions fitted to the responses of individual participants are shown in Fig. 3. Distractor effects were significantly greater during one-handed compared with two-handed touch, F(1,16) = 12.299, *p* = 0.003, $\:{\eta\:}_{p}^{2}$ = 0.435. Slope gradients relating target responses to distractor groove width were significantly different from zero for both the comparison-finer, t(16)=4.293, *p* < 0.001, d = 1.041, and comparison-coarser, t(16) = 6.366, *p* < 0.001, d = -1.544, targets during one handed touch. As distractor groove width increased, targets in the comparison interval were more frequently identified as rougher than standard targets. In contrast, slopes in the two handed conditions were no different from zero, t(16)=1.309, *p* = 0.209, for comparison-finer and t(16) = 1.793, *p* = 0.92, for comparison-coarser conditions.

### Intercept analysis

Figure 3 shows the y-0 intercept of linear functions fitted to the responses of individual participants. Intercepts show predicted roughness responses to targets paired with distractors of the same groove width. There was no effect of the number of hands used to feel the surfaces, F(1,16) = 0.002, *p* = 0.961. However, there was a significant effect of varying-interval, F(1,16) = 5.996, *p* = 0.026, $\:{\eta\:}_{p}^{2}$ = 0.273, and a significant interaction between varying-interval and the number of hands used to touch the surfaces, F(1,16) = 4.797, *p* = 0.044, $\:{\eta\:}_{p}^{2}$ = 0.231. In the one handed condition, coarser (1200 μm) targets were judged as the rougher surface more often than the finer (1160 μm) targets, t(16)=3.473, *p* = 0.002, d = 0.842; intercept for the coarser target (mean = 0.56, sd = 0.09) versus finer target (mean = 0.42, sd = 0.09). In contrast, there was no evidence of a difference in intercept when the target and distractor were on opposite hands; coarser target intercept (mean = 0.51, sd = 0.12) and fine target intercept (mean = 0.47, *p* = 0.11), t(16)=0.995, *p* = 0.167.

## Discussion

In the present 2IFC study, roughness discrimination of target gratings touched with the right hand thumb was assessed as a function of the coarseness of distractor gratings touched simultaneously with the index finger of the same hand or the index finger of the opposite hand. When the thumb and index finger were on the same hand, roughness responses varied systematically with the relation between target and distractor groove width. As the groove width of the distractor presented with a target increased, the proportion of rougher response to that target also increased. This resulted in positive slopes for psychometric functions relating target responses and distractor groove width. While the magnitude of distractor effects was similar for coarser (1200 μm) and finer (1600 μm) targets, coarser targets were judged as rougher more frequently than finer targets at all levels of distractor groove width indicating participants responded to the target as well as distractor surfaces. Together the slope and intercepts suggest both target and distractor surfaces contribute to perceived roughness at the target thumb. Similar findings were not evident in two-handed touch. There was no consistent pattern in the relationship between distractor groove width and roughness responses to target gratings. In addition, two handed roughness judgments did not distinguish between coarser and finer targets even though they were reliably discriminated in the one handed condition.

The effect on roughness judgments at a target digit of distractor surfaces presented to another digit on the same hand extends previous findings of Kahrimanovic et al.^4^ using embossed Braille dots with 1.3–2.3 mm spacing, and Roberts and Humphreys^3^, using sandpaper with 0.04–0.19 mm grit size. Kahrimanovic and colleagues^4^ used the index and middle finger on one hand in showing 2IFC roughness judgments at one digit were affected by the roughness of a distractor presented to the other digit. Participants in Roberts and Humphreys^3^ used a precision touch involving index finger and opposed thumb, similar to the present study. In a subsequent study, Roberts^5^, 2IFC roughness judgments at the thumb were also found to be affected by the roughness of a distractor presented to the index finger, either of the same or the opposite hand. However, in contrast to the present study, the effect reported by Roberts^5^ was the same whether thumb and index finger were on the same or different hands.

The present 2IFC results support an integrative account of interactions between roughness sensations arising at separate digits on the same hand. Distractor roughness at the index finger was not only difficult to ignore, but systematically changed roughness perception at the attended thumb. However, these data do not support integration of touch signals from digits from opposite hands. In the present study only the 1200 μm and the 1160 μm gratings were touched by the participant’s thumb. Participants were not informed that the same pair of gratings would be presented to the thumb for discrimination on each trial. On completion of testing the participants were questioned about the level of difficulty of the task. All reported the discrimination task difficulty as varying over trials. On being informed that the same pair of gratings was being presented to the thumb on each trial, none of the participants reported being aware of this during testing. Experiment 2 addresses the effect of distractors on perceived roughness more directly, asking participants to report the roughness magnitude of sensations at their target thumb while ignoring sensations at their distractor index finger.

## Experiment 2: Roughness Estimation

Experiment 2 was designed to test whether distractor effects observed using the 2IFC roughness discrimination task in Experiment 1 would extend to the experience of roughness magnitude measured using an absolute magnitude estimation task. Are roughness magnitude judgments of a grating presented to the thumb affected by simultaneous presentation of a distractor grating to a second digit on the same hand alone or would distractor effects extend across opposite hands? Under these conditions, are roughness judgments of relatively smooth targets “pulled up”, i.e. increased in magnitude, by rougher distractors and those of relatively rough targets pulled down by smoother distractors relative to judgments of the targets presented in isolation? And, if this effect occurs, is it more pronounced when the target and distractor are presented to digits on the same hand compared with different hands?

### Method

#### Participants

Thirteen participants took part in this experiment. None of these participants had taken part in Experiment 1. All were classified as strongly right-handed (mean laterality index of 96.5)^6^, reported normal tactile sensation and normal or corrected-to-normal vision. Their ages ranged from 19 to 39 years, with a mean age of 25±6.5 years. Seven of the participants were female. All gave informed consent and received £18 sterling for their participation in three 1 h sessions.

#### Stimuli

Participants judged the roughness of Tufset square wave gratings described in Experiment 1. The gratings used in Experiment 2 had groove widths of 330, 420, 880, 960, 1120, 1240, 1360 and 1520 μm. Gratings with groove widths greater than 420 μm had ridge widths of 400 μm while the ridge width of the 330 μm and 420 μm gratings was 100 μm. Gratings were presented as either a single stimulus, where one grating was attached to a mounting card, or a double stimulus pair where a grating was attached to each side of the mounting card as in Experiment 1.

#### Procedure

Testing took place in 3 sessions with each session conducted on separate days. A method of absolute magnitude estimation (AME)^2,14,15^ was used to measure participants’ subjective sense of roughness. AME involves reporting the magnitude of a sensation by assigning it a number of the same psychological magnitude. No standard or modulus was used, and the participants were free to assign any subjective impression of roughness they felt was appropriate. At the start of their first experimental session participants practiced using AME to report the subjective lengths of lines. They then completed two blocks of trials in which they judged the roughness of touched gratings. The two subsequent sessions consisted only of roughness judgments of gratings.

### Line judgment task

Participants sat at a table facing a 24 inch computer monitor in a semi-dark room. The distance between the screen and each participant’s head was about 72 cm. The following instructions, previously used by Gescheider and Hughson (1991), were given for the line judgment task.

“You have impressions of what is a long-, short-, and medium-length line. You also have impressions of what is a large-, small- , and medium- size number. When I present a line on the screen, I would like you to assign a number to it so that your impression of the size of the number matches your impression of the length of the line. Do not try to measure the line or compare its length with those of other lines. Just try to look at each line and say a number that seems right for it. You can use any positive number that you want to. You can use decimals or fractions if you feel they are appropriate.”^14^.

A white horizontal line measuring either 0.4, 1.5, 2, 4, 8 ,16–20 cm in length and 0.4 cm in width was shown on a black background in a darkened room. Each of the lines was presented 4 times, except for 0.4 and 1.5 cm lines which were presented 3 times for 9 participants, in a random order. An empty black screen, lasting 2 s appeared at the start of each trial. The line was then presented for 1 s and was replaced by the black screen. Two seconds later a white question mark appeared on the screen prompting participants to give a verbal judgment of each line length. The next trial was initiated by a press of the space bar on the keyboard.

### Roughness judgment task

On completion of the line judgement task participants were instructed to use the AME method to report their impression of the roughness of gratings touched using their thumb. The roughness AME instructions were the same as those used for the line-judgment task. However, instructions were given to match impressions of the size of numbers with impressions of the roughness of the surfaces.

Participants were seated at a table and a blindfold was placed over their eyes at the start of testing so that participants did not see the grating surfaces. Roughness perception was measured in 3 sessions carried out on 3 separate days. There were 2 blocks of roughness trials in each session. Testing began with judgments of single gratings followed by a block of double gratings trials. This pattern occurred twice in each experimental session. The 8 single gratings were presented twice in a randomised order. During single grating trials the participant started with their right hand resting on the table. The grating was placed on the table at a position previously indicated to the participant by the experimenter. The non-grating surface of the mounting card rested on the table and the grating faced upward. The experimenter held the mounting card in place. Following an experimenter touch to the back of the hand the participant moved their hand to the stimulus position. They then ran their right thumb over the grating by moving the hand towards the body. Instructions were given to move the thumb along the grating in one fluid movement at a comfortable scanning speed. The movement was stopped when the thumb no longer contacted the grating surface. The participant then gave a verbal report of their roughness rating for that surface.

A block of double gratings trials followed the single grating testing. Each of the 8 grating groove widths was paired with each of the 7 other gratings of a different groove width. Thus, there were 56 different pairs of double grating stimuli (grating pairs). Instructions were given to focus attention on the grating felt using the right thumb (the target grating) and to ignore the grating felt by the other digit (the distractor grating). All 56 combinations of target and distractor groove width were presented at least once in each block of trials. Combinations involving 420, 1120 and 1360 μm groove width targets were presented twice in each block. These targets, with increased sampling, were included to allow an extended analysis of the effects of distractors. In total there were 77 trials in each double grating block. The different target and distractor combinations were randomly interleaved in each block. As in Experiment 1, the grating pairs were explored using the right thumb and right index finger (one-handed condition) or the right thumb and left index finger (two-handed condition). Three blocks of single and double grating trials were completed for each digit combination. Eight participants completed the one-handed blocks first and five completed the two-handed blocks of trials first. The instructions and feedback concerning movement used to scan the gratings was the same as Experiment 1.

#### Data analysis

Roughness ratings for each target-distractor combination for each participant were found by calculating the geometric mean across repeated ratings. This was done separately for each hand condition. These ratings were normalized within participants by dividing each mean rating by the participant’s grand mean across all conditions. The data of one participant was excluded due to failure to comply with magnitude rating instructions regarding positive values.

Roughness magnitude ratings for the different gratings, averaged over distractors, are first examined to confirm, in line with previous reports, that subjective roughness of these gratings increased with increasing groove width. Next, distractor effects on subjective roughness ratings are reported for 420, 1120 and 1360 μm groove width targets where extended sampling was conducted.

The normalised ratings, averaged over distractors, were analysed using a repeated measures ANOVA with number of touching digits (single-digit [target only] or two-digit [target and distractor]), hand condition (one-handed or two-handed block) and target grating groove width (330, 420, 880, 960, 1120, 1240, 1360 and 1520 μm) as within-subjects factors.

To determine whether there was a significant interaction between the effects of distractor groove width and the hand touching the distractor on the perceived roughness of target gratings, ratings for the most sampled targets: 420 μm, 1120 μm and 1360 μm were examined when paired with distractor gratings common to all 3 targets (330, 880, 960, 1240 and 1520 μm gratings). These data were analysed using a repeated measures ANOVA, with target groove width, distractor groove width and hand (one-handed or two-handed touch) as within-subjects factors.

## Results

### Effects of spatial period on subjective roughness

**Fig. 4:**
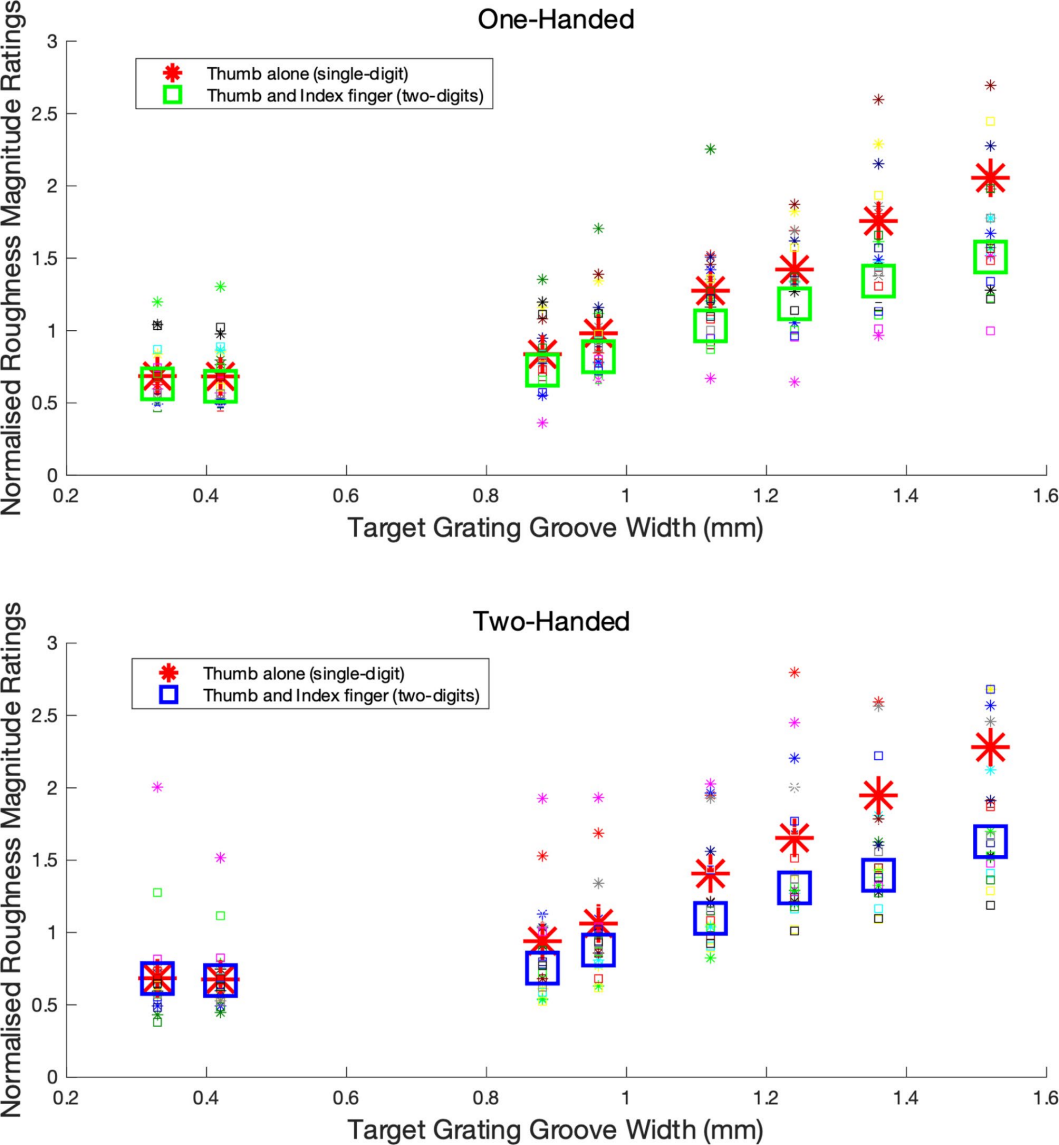
Normalised roughness ratings of target square wave gratings touched using the thumb alone or the index finger and thumb together. Individual participants ratings are shown by small symbols and group means by larger bold symbols (stars for single-digit touch and squares for two-digit touch which has been averaged over distractor groove widths). Data from one-handed conditions are shown in the top panel and two-handed conditions in the bottom panel.

Normalised roughness magnitude ratings when touching using the thumb alone (single-digit condition) and using the thumb and index finger (two digits: one-handed or two-handed) are shown in Fig. 4. The ratings for each target grating in the two-digit conditions have been averaged over the different distractor stimuli. Ratings in one-handed conditions are shown in the top panel of Fig. 4 and those from the two-handed conditions in the bottom panel. The figure shows roughness ratings increased with target groove width.

Ratings of surface roughness increased with increasing target groove width, F(7,77) = 76.315, *p* < 0.001, $\:{\eta\:}_{p}^{2}$ = 0.874 and were greater when touching using the thumb alone (single digit) compared to using the thumb and index finger (two-digits) together, F(1, 11) = 30.806, *p* < 0.001, $\:{\eta\:}_{p}^{2}$ = 0.737. The significant interaction between these factors, F(7,77) = 22.079, *p* < 0.001, $\:{\eta\:}_{p}^{2}$ = 0.667, reflects an increasing difference between thumb only and thumb plus index finger (i.e. target and distractor) conditions with increasing grating groove width. There were no significant effects involving the number of hands used.

### One-handed vs. two-handed effects: distractor effects on roughness ratings

**Fig. 5:**
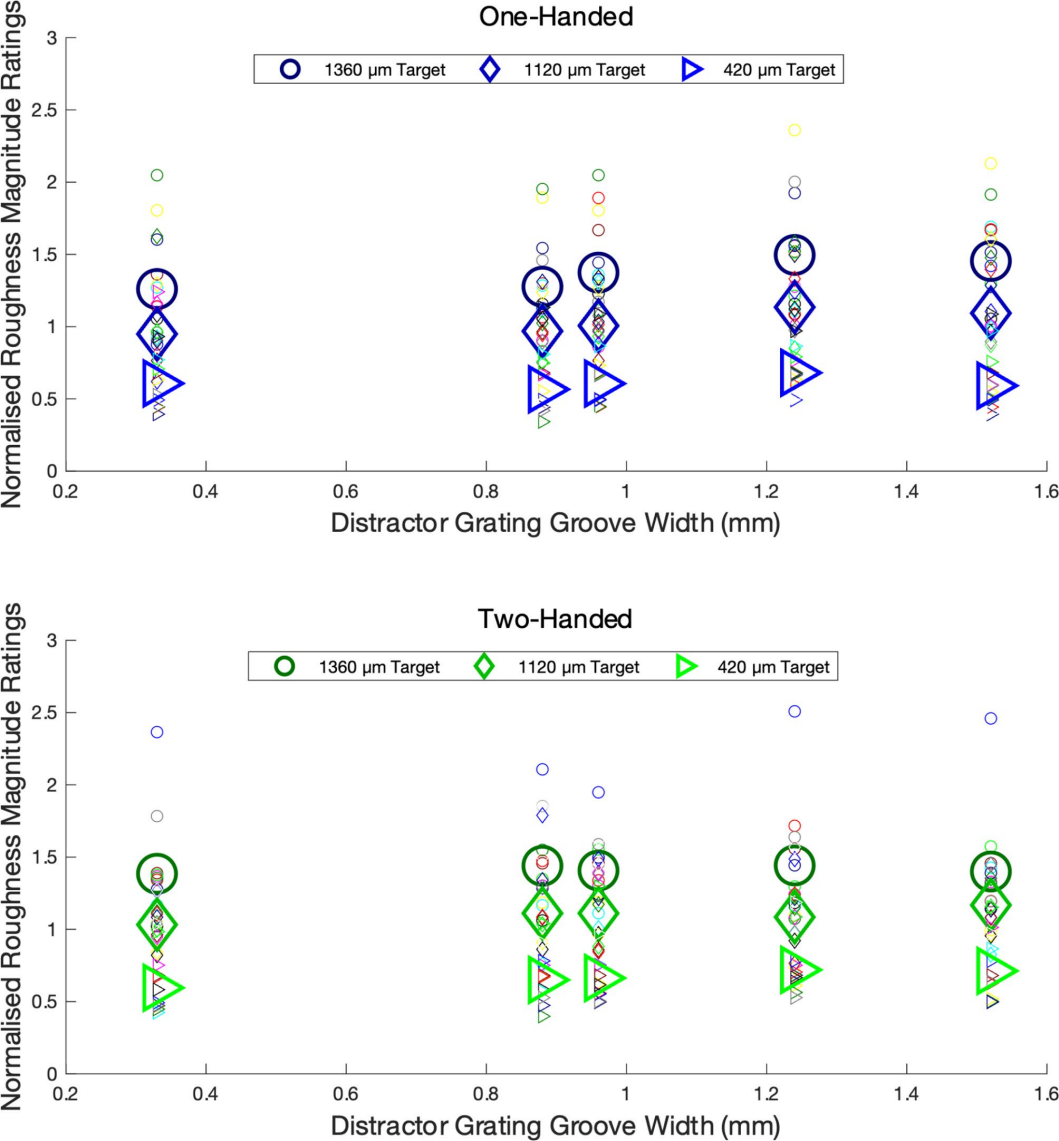
Roughness ratings for fine (420 μm GW, triangles), medium (1120 μm GW, diamonds) and coarse (1360 μm GW, circles) target gratings when paired with 5 different distractor gratings (GWs: 330, 880, 960, 1240 and 1520 μm). These distractor gratings were common to all 3 targets. Individual participants ratings are shown by small symbols and group means by larger bold symbols. One-handed touch is shown in the top panel and two-handed performance is shown in the bottom panel.

Distractor effects on roughness magnitude ratings are shown in Fig. 5. As expected, roughness ratings increased with increases in target groove width, F(2,22) = 64.105, *p* < 0.001, $\:{\eta\:}_{p}^{2}$ = 0.854. Roughness ratings also increased with distractor groove width, F(4,44)= 7.810, *p* < 0.001, $\:{\eta\:}_{p}^{2}$ = 0.415. There was no main effect of hand on reports of subjective roughness, F(1,11)= 0.308, *p* = 0.590 nor any significant interactions between all of the factors.

As shown in Fig. 4, roughness ratings for the 6 most coarse gratings increased monotonically with groove width. However, this pattern did not extend to the 330 μm and 420 μm targets gratings. It is notable that this pair of fine gratings are very close in groove width to each other but distant from the six coarse gratings. In addition, due their small groove width, roughness perception of these fine gratings is likely to rely on vibratory cues alone. This contrasts with the coarse surfaces where both spatial and vibratory cues would be available^7,16,17^. A further, exploratory, ANOVA was carried out, restricting the analysis to those targets (1120 and 1360 μm) and distractors (880, 960, 1240 and 1520 μm) falling clearly in the coarse category, where surface detail could be perceived by both sliding and pressing contact. This analysis revealed effects of target groove width, F(1,11) = 73.254, *p* < 0.001, $\:{\eta\:}_{p}^{2}$ = 0.869 and distractor groove width, F(3,33)= 5.602, *p* = 0.003, $\:{\eta\:}_{p}^{2}$ = 0.337 on the ratings of target roughness. There was also a significant interaction between hand and distractor, F(3,33)= 3.019, *p* = 0.044, $\:{\eta\:}_{p}^{2}$ = 0.215. Separate ANOVA’s for the one-handed and two-handed conditions showed that in addition to the anticipated higher ratings for 1360 compared with 1120 targets (one-handed touch, F(1,11) = 38.184, *p* < 0.001, $\:{\eta\:}_{p}^{2}$ = 0.776, and two-handed touch, F(1,11)= 40.916, *p* < 0.001, $\:{\eta\:}_{p}^{2}$ = 0.788) there was an effect of distractor groove width on target ratings with one-handed, F(3,33)= 6.533, *p* = 0.001, $\:{\eta\:}_{p}^{2}$ = 0.373, but not two-handed, F(3,33)= 0.275, *p* = 0.853, sliding contacts.

## Discussion

The results of Experiment 2 are consistent with previous findings that perceived grating roughness increases with spatial groove width^10–13^. They also extended the findings from the first study in showing interaction between digits with AME, as opposed to 2IFC discrimination, as the response measure. In contrast to 2IFC, AME addresses perceived roughness directly. AME also avoids the requirement, inherent to 2IFC experimental designs, to hold a representation of the first stimulus in memory for comparison with the second stimulus presented 1–2 s later. Consistent with the first study, this second study showed perceived roughness at the thumb is increased by a rougher stimulus and decreased by a smoother stimulus presented simultaneously to the index finger. Slopes of curves for two-digit contact were shallower than those for one-digit contact suggesting the roughness of fine surfaces is pulled up by their predominately coarser distractors and the roughness of coarse targets pulled downward by their predominately finer distractors on a second digit.

Overall, the pattern of results in Experiment 2 is consistent with the observations in Experiment1 of distractor effects in one handed touch but no evidence of similar effects when touching with two hands. Analysis of the full AME dataset failed to show an effect of different hands on distractor effects. However, an exploratory analysis, using a subset of the data created by omitting the finest target (420 μm) and distractor (330 μm) gratings, a reliable distractor effect was found in the one-handed but not the two-handed condition. This, admittedly post hoc, analysis showing sensitivity to distractor gratings in the one-handed but two-handed condition suggests that the hand effect observed in the first study is not attributable to differences in task demands between the AME and 2IFC paradigms.

Roughness ratings were lower when touched with two digits compared with a single digit. This finding contrasts with that of Verillo and colleagues^2^ who, using AME of roughness with sandpaper stimuli, found greater roughness ratings with two digits compared with single finger contact. One possible explanation for the difference in findings is that Verillo et al.^2^ did not instruct participants to attend to one stimulus and ignore the other. Thus, their participants may have understood that they should attempt to integrate their perception of roughness across the digits. In the present study participants were explicitly told to ignore the distractor target. Integrative versus comparative perceptual goals have been shown to affect cortical responses to tactile stimuli on pairs of fingers. For instance, instructions to compare the movement directions of tactile stimuli on separate fingers have been shown to result in suppressive cortical activations compared with when instructions were given to combine the two stimuli and report their average direction^18^. Iguchi et al.^19^ observed task dependent changes in S1 cortical responses to vibrotactile stimuli on the index and middle fingers. There was increased cortical differentiation of the index and middle fingers of one hand when participants identified the stimulated finger. In contrast, representations of the two fingers were indistinguishable when differentiation was not required, i.e. when participants experienced the stimuli passively or reported stimulus frequency.

Another possible account of the increased roughness ratings in the single digit condition is that, in this condition, participants may have pressed harder in the single-digit condition because of the different way in which the stimulus was explored. Participants pressed down with the thumb on gratings placed on the table in the single digit condition, but pressed their thumb against their index finger in two digit conditions. Thus, the observed difference in roughness ratings in one-digit and two-digit conditions might, in part, reflect differences in normal force when sliding across the surfaces. In future active touch experiments it will be important to measure or control contact forces. We know that perceived roughness is affected by force^10,20,21^. But, more recently, it has also been shown that participants actively control normal and tangential forces to optimise roughness signals^7^.

## General Discussion

Roughness, a dimension of surface texture^22^, is an important attribute of an object. It affects both perception, for example, discriminating real wood from laminate, and action, as illustrated by a rough surface tending to afford a higher friction contact for a more secure grip see^23 for a review^. People often explore the surface textures of objects and fabrics using sweeps of multiple digits or with fingers and thumb opposed^1^. However, little is known about whether and how the brain combines information from multiple touch sources to generate an overall sense of the texture of touched surfaces.

Previously Kahrimanovic and colleagues^4^ studied roughness discrimination using a pair of neighbouring digits in planar touch on dot patterns. Roberts and Humphreys^3^ used pairs of digits from the same hand in precision grip touch on sandpaper surfaces. In both sets of studies roughness discrimination at an attended digit was found to be affected by surface texture at a non-attended (distractor) digit. This interaction between digits has also been observed when the two digits are on different hands^5^. The two experiments reported in this paper examined whether the integrative patterns of behaviour previously reported when selectively attending to the roughness on one of two digits would occur both in a discrimination task, measuring perceived roughness indirectly, and in a magnitude estimation task, where subjective report was directly measured. Perceived roughness of square wave gratings was measured using a 2IFC discrimination task in Experiment 1 and an AME task in Experiment 2. In both tasks attention was directed towards a target digit (the thumb) and away from the distractor digit (the index finger). The results of the present experiments confirm previous findings that the perception of roughness at a target digit is influenced by the roughness at distractor digits. This effect was observed for digits on the same hand, consistent with Kahrimanovic et al.^4^, and Roberts & Humphreys^3^, but contrasts with an effect for digits across both hands, as reported by Roberts^5^.The present study also extends the findings of those studies using dot-patterns and abrasive surfaces to non-abrasive square wave surfaces and the magnitude estimation study of Verillo and colleagues^2^ to a selective attention paradigm.

Together, results from the two experiments suggest that interactions between the digits for roughness perception occur irrespective of the differences in task demands between the two psychophysical methods and the requirement to selectively attend to one digit. However, the present experiments show no effect between the hands which contrasts with Roberts^5^ possibly reflecting the different types of surfaces used in the two experiments. The abrasive nature of sandpaper surfaces may affect, not only how surfaces feel, but also how such surfaces are touched and the condition of the surface of the skin, in turn affecting the transmission of roughness cues. It is also worth noting that roughness is a material attribute of surfaces and that surfaces fitting within the grasp of a hand often relate to a single object, while those touched by the opposite hands may more often relate to separate objects. In the Roberts^5^ study the sandpaper (target and distractor) surfaces, attached to either side of the same card, produced a stimulus that was less than 2 mm thick, contrasting with the present study where the combination of target and distractor produced a 13.5 cm thick stimulus. Knowledge of whether touch sensations originate from a single as compared with two objects has been shown to change the distribution of tactile spatial attention. It is more difficult to withdraw attention from opposite hand stimuli when they appear to come from the same compared with separate objects^24–26^. When actively touching and judging the roughness of surfaces, the extent to which those surfaces are perceived as belonging to a single object, and the consequences this may have on filtering out of distractors in one-handed compared with two handed touch, are questions for future research,

The finding of differences in tactile processing within compared with between the hands is consistent with previous reports of hand identity affecting interactions between digits in behavioural^27–30^ and electrophysiological studies^31^ beyond roughness and may reflect a more general constraint on the processing of touch with the hands. For example, Tamè and colleagues observed stronger vibrotactile masker effects within than between the hands, a finding also reported when discriminating movement direction across the skin^28,29^.The present results also reveal stronger distractor effects for digits on the same versus different hands. In contrast, Arslanova and colleagues^30^ found greater precision judging the average movement direction of touch across digits on two hands compared to one hand. However, it is worth noting their participants were required to combine rather than differentiate sensory signals from the two fingers.

In summary, the results here support previous findings that it is difficult to restrict processing of surface roughness to a single digit within one hand. They also add to a body of evidence based on experiments using different stimuli and tasks, suggesting that hand identity affects interactions across the digits. However, it is important to be aware that, even for the same type of stimuli, the type of interaction between digits can also depend on which feature is task relevant^8^.

## Data Availability

The datasets generated during and analysed during the current study are available in the OSF repository, https://osf.io/nyx7s/?view_only=ad089d6ef1c04ffe9d2308816d97ec0f.
